# Diapause-like Drug-Tolerant Persister State: The Key to Nirvana Rebirth

**DOI:** 10.3390/medicina60020228

**Published:** 2024-01-28

**Authors:** Han-Lin Chen, Wei-Lin Jin

**Affiliations:** 1 The First Clinical Medical College, Lanzhou University, Lanzhou 730000, China; chenhl2021@lzu.edu.cn; 2 Institute of Cancer Neuroscience, Medical Frontier Innovation Research Center, The First Hospital of Lanzhou University, Lanzhou 730000, China

**Keywords:** cancer relapse, diapause, dormancy, drug-tolerant persister, drug resistance

## Abstract

Cancer is one of the leading causes of death in the world. Various drugs have been developed to eliminate it but to no avail because a tumor can go into dormancy to avoid therapy. In the past few decades, tumor dormancy has become a popular topic in cancer therapy. Recently, there has been an important breakthrough in the study of tumor dormancy. That is, cancer cells can enter a reversible drug-tolerant persister (DTP) state to avoid therapy, but no exact mechanism has been found. The study of the link between the DTP state and diapause seems to provide an opportunity for a correct understanding of the mechanism of the DTP state. Completely treating cancer and avoiding dormancy by targeting the expression of key genes in diapause are possible. This review delves into the characteristics of the DTP state and its connection with embryonic diapause, and possible treatment strategies are summarized. The authors believe that this review will promote the development of cancer therapy.

## 1. Introduction

Cancer, which induces a massive global economic cost and health burden [1], is a major public health problem worldwide [2]. Although cancer is extremely harmful, people are often at their wits’ end when faced with it. A small number of tumors will always remain after each treatment. In fact, they can go dormant to evade therapy, which has become an important problem which cannot be ignored in cancer therapy [3]. During tumor dormancy, cancer cells remain but stop progressing and lose sensitivity to treatment [4]. People have spent decades studying the mechanism of tumor dormancy, hoping to change the dilemma of cancer therapy. The mainstream theory is that tumor dormancy is caused by the cessation of proliferation of cancer cells (cellular dormancy) [5] or the maintenance of a balance between apoptotic cells and proliferating cells (tumor mass dormancy) [3]. Despite this, the scientific community still has not truly confirmed the mechanism of tumor dormancy, let alone proposed a medical method which utilizes the mechanism of tumor dormancy to completely cure tumors.

Recently, a new theory to explain tumor dormancy has emerged: the drug-tolerant persister (DTP) state. This theory has greatly stimulated interest in exploring the mystery of tumor dormancy. To be exact, some tumor cells can survive after the implementation of therapy, and most of them are in a reversible quiescent state [6]. However, scholars have not been able to clarify the specific mechanism of the DTP state. Polyploid giant cancer cells (PGCCs) play an important role in tumor dormancy and recurrence and may be the culprit in tumor drug resistance [7], which may be related to the formation of a DTP state. Understanding of the formation of DTP state cancer may become an important breakthrough in the mechanism of tumor dormancy.

In a study of colorectal cancer cells, Rehman et al. confirmed that the DTP state is transcriptionally and functionally like diapause [8]. Specifically, diapause may be the mechanism of the DTP state. Diapause is a survival strategy of animals that deals with poor living conditions through developmental pauses and is reactivated after the living conditions have been restored [9]. In the process of diapause, animals become long-lived, stress-tolerant, stagnant, and unable to reproduce [10]. A recent study on acute myeloid leukemia re-emphasized the link between embryonic diapause and the DTP state [11]. The discovery of a diapause-like DTP state is expected to explain the real mechanism of tumor dormancy and cause a turnaround in tumor therapy.

**Figure 1:**
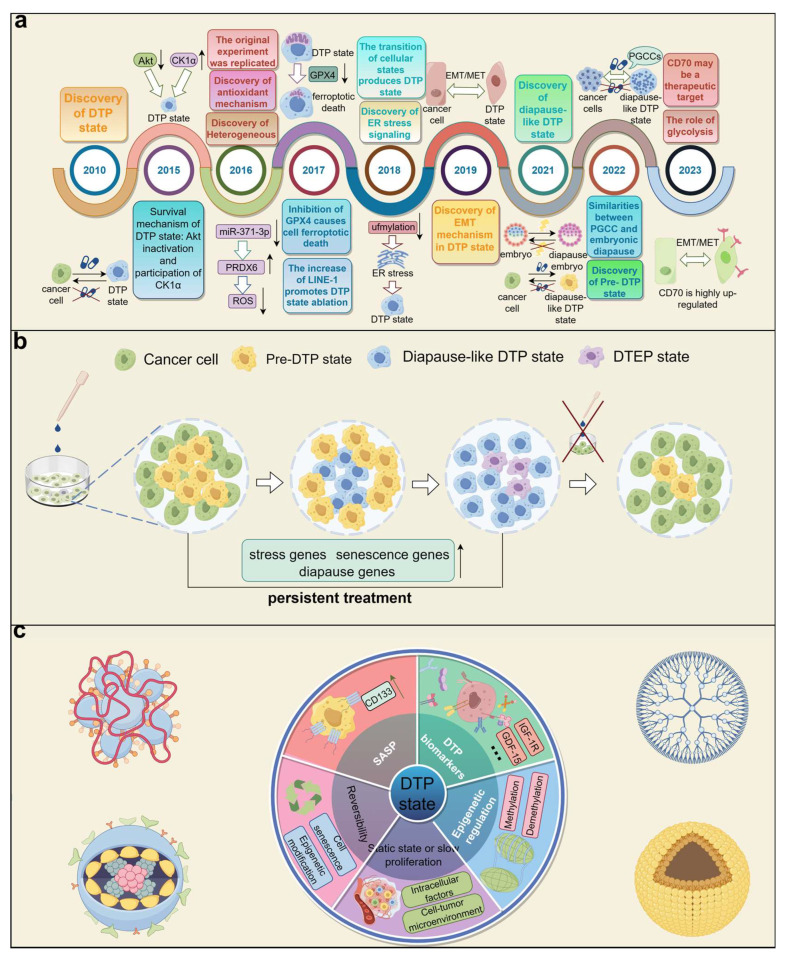
Introduction of a drug-tolerant persister (DTP). (**a**) History of DTP theory. Since the DTP state was discovered in 2010, DTP theory has been continuously improved. The discovery of the diapause mechanism in 2021 has greatly promoted the development of DTP theory. In the last 3 years, new achievements have continued to emerge. Diapause-like DTP theory is expected to become the mainstream theory of tumor dormancy. (**b**) Cyclic process of the DTP state. Before drug treatment, a very small number of cancer cells are in a pre-DTP state. After drug treatment, more cancer cells enter the pre-DTP state and then go into the diapause-like DTP state, and about 20% of the cells in the diapause-like DTP state enter the DTEP state. Until the treatment is stopped, the subgroup composition of the tumor cell group is restored. (**c**) Characteristics of the DTP state. By understanding the characteristics of DTP cells, we may be able to find a solution to this challenge and even achieve a complete cure for cancer patients. Akt, protein kinase B; CK1α, Casein kinase 1α; PRDX6, Peroxiredoxin 6; GPX4, Glutathione Peroxidase 4; LINE-1, Long interspersed nuclear elements 1; ER, endoplasmic reticulum; EMT, epithelial-to-mesenchymal transition; MET, mesenchymal-to-epithelial transition; PGCCs, polyploid giant cancer cells; CD133, Prominin-1; IGF-1R, insulin-like growth factor 1 receptor; GDF-15, Growth differentiation factor 15; SASP, senescence-associated secretory phenotype. “↑” indicates upregulation, and “↓” indicates downregulation.

Although people have met the concept of a diapause-like DTP state, its value has not been fully tapped. This article reviews the concept, characteristics, and possible treatment strategies of the diapause-like DTP state to explore its value in tumor treatment and contribute to the overcoming of the problem of refractory tumors (Figure 1).

## 2. Characteristics of the DTP State

The theory of the DTP state is still in its primary stage of development. A clear understanding of the characteristics of the DTP state will help make its concept distinct and promote the development of DTP theory. The authors will elaborate on the main characteristics of the DTP state.

### 2.1. Static State or Slow Proliferation

Resting state and slow proliferation are the most prominent characteristics of the DTP state, which means that some cancer cells stay in the G0 phase or enter the cycle of slow proliferation to escape the cytotoxic stress of targeted therapy [6]. This state of quiescence or slow proliferation is temporary, which is different from the quiescence or slow proliferation of normal cells. This feature connects the DTP state with the phenomenon of tumor dormancy, which makes the DTP state the key to solving the mystery of tumor dormancy. 

In stem cells, the level of mitochondrial autophagy determines whether cells remain static or experience activation [12]. Similarly, PTEN-induced putative kinase 1 (PINK1)-mediated mitochondrial autophagy may contribute to the initiation of a DTP state by promoting mitochondrial homeostasis. The activation of PINK1 is one of the important contents of mitosis. Phosphorylated PINK1 participates in the regulation of mitosis by acting as a monomer ubiquitin kinase and a Parkin Ubl kinase [13]. Research shows that the downregulation of Myc weakens the transcriptional inhibition of PINK1, which induces PINK1 expression and regulates mitochondrial autophagy, thus promoting the production of a DTP state [14].

The formation of the slow proliferation state is the result of the interaction between the cell itself and the environment. Cell-intrinsic epigenetic reprogramming, such as lysine demethylase (KDM)6, promotes the slow cycle. At the same time, the growth in tumor microenvironment (TME) fibroblasts and inflammatory mediators may affect the slow proliferation of cells [15]. Current research shows that increased infiltration of Ccr2+ macrophages in the TME can promote the formation of a DTP state [16]. Increased Secreted Frizzled-Related Protein 1 (SFRP1) secreted by fibroblasts can help cancer cells escape from the slow proliferation state and, vice versa, can promote the slow proliferation state [17].

While most cancer cells in a DTP state are in a dormant state, approximately 20% of them eventually resume normal proliferation in the presence of a drug, producing cell colonies known as “drug-tolerant extended persisters” (DTEPs), which can proliferate indefinitely in the presence of the drug [6]. Cells in this state are remarkably harmful.

Many factors lead to the static state or slow proliferation state of the DTP state. The actual mechanism is considerably more complex than what has currently been discovered.

### 2.2. Reversibility

Reversibility is also one of the important characteristics of the DTP state [18]. When a drug is discontinued, the tumor cells will regain sensitivity to the drug [19]. In other words, unlike the clonal evolution driven by gene mutations, the clonal complexity of recurrent tumors does not disappear during chemotherapy. Therefore, the difference between the DTP state and cancer stem cells is that no gene mutation occurs [20]. It strongly implies that regulation by nongenomic or epigenomic mechanisms is involved in this reversibility [21]. As a result, the reversibility of the DTP state can be studied from the perspective of epigenetics.

In addition, the reversibility of the DTP state is related to cellular senescence. The DTP state displays hallmarks of cellular senescence [22], and senescent cells have a definite impact on cancer recurrence and spread. Experiments have found that removing senescent cells after chemotherapy can prevent or delay cancer recurrence and spread to distant tissues [23]. Cellular senescence may also be one of the mechanisms leading to the reversibility of the DTP state. What is gratifying is that there are a number of drugs that have a specific cytotoxicity to senescent cells, which may help to avoid the drug resistance capabilities of cancer cells [24].

**Figure 2:**
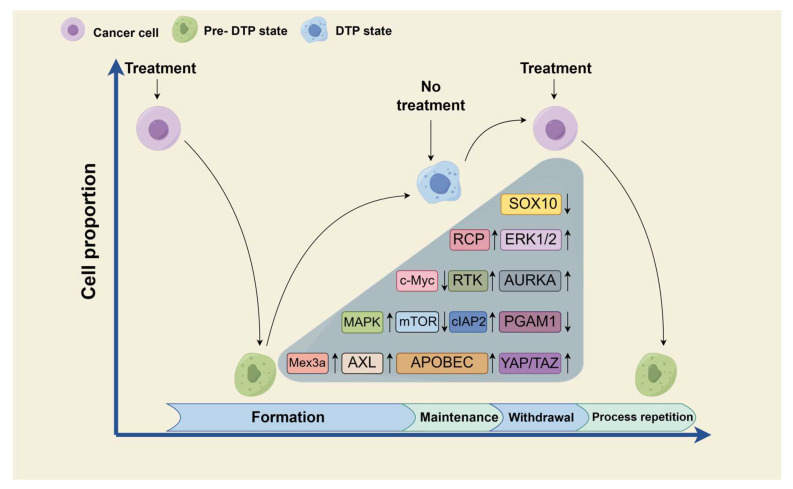
Partial mechanism of the reversibility of the DTP state. Reversibility is an important therapeutic goal of the DTP state that is worthy of our in-depth understanding. Here, we list some signal molecules that may be involved in reversibility. SOX10, sex-determining region Y-box 10; RCP, Rab coupling protein; ERK1/2, extracellular signal-regulated kinase 1/2; c-Myc, cellular-myelocytomatosis viral oncogene; RTK, receptor tyrosine kinase; AURKA, aurora kinase A; MAPK, mitogen-activated protein kinase; mTOR, mammalian target of rapamycin; cIAP 2, cellular inhibitor of apoptosis protein 2; PGAM1, phosphoglycerate mutase 1; Mex3a, Mex3 RNA-binding family member A; AXL, anexelekto; APOBEC, apolipoprotein B mRNA-editing enzyme catalytic polypeptide-like; YAP, Yes-associated protein; and TAZ, transcriptional coactivator with PDZ-binding motif. “↑” indicates upregulation, and “↓” indicates downregulation.

The generation of this reversibility is also related to the changes in many other signal molecules, which are summarized in this paper (Figure 2).

### 2.3. DTP Biomarkers

Various biomolecules related to the DTP state that have been discovered thus far may become biomarkers of the DTP state. Identifying biomarkers is helpful for finding and identifying the DTP state to establish correct treatment strategies.

Growth and differentiation factor 15 (GDF-15) belongs to the transforming growth factor-β (TGF-β) superfamily of proteins, and it plays a role in the pathogenesis of tumors [25]. The expression/secretion of GDF-15 can only be detected in a DTP state, and GDF15 is not expressed or secreted in sensitive cells (parents) and completely resistant cells [26]. These findings validate the presence of GDF-15 as a biomarker of the DTP state.

The DTP state can also be found by detecting key biomolecules related to the signaling pathways that activate a DTP state, so these key biomolecules have the potential to become DTP biomarkers. A luminal-like DTP state survives via estrogen receptor-dependent induction of serum/glucocorticoid-regulated kinase family member 3, leading to the rewiring of the PI3K/AKT/mTORC1 pathway to enable AKT-independent mTORC1 activation; thus, mTOR can be used as a candidate DTP biomarker [27]. In patients with residual disease treated with a circulating DTP state and EGFRi, growth arrest-specific protein 6 (GAS6) has been observed to be upregulated, while ablation of the GAS6 receptor AXL eliminates drug resistance [28]. Therefore, the activation of the GAS6-AXL pathway is related to the DTP state, and GAS6 or AXL may become biomarkers of the DTP state.

Acetylcholine (ACh) is well recognized as a neurotransmitter. It is a ubiquitous signaling molecule produced by a wide range of non-neuronal cell types [29]. The neurotransmitter ACh has been found to accumulate specifically in DTP states via metabonomics and transcriptomics, and EGFR-TKI treatment increases the expression of choline acetyltransferase, the rate-limiting enzyme in ACh biosynthesis mediated by YAP. In vitro, in vivo, genetic, and pharmacological modulation of ACh production or ACh signal transduction can predictably modulate the degree of DTP state formation [30]. As a result, Ach is another biomarker option.

Three proteins are highly expressed in the DTP state of high-grade ovarian carcinomas: carcinoembryonic antigen-related cell adhesion molecule 6, crystallin alpha B, and SRY-box transcription factor 2 [31]. They may become biomarkers for predicting disease resistance and recurrence. In addition, developmental pluripotency-associated 3, aldehyde dehydrogenase, CD44, CD133, CD271, ABCB5, and KDM5B all show potential to become DTP biomarkers [32,33]. They may be helpers for further studies of the DTP state in the future.

### 2.4. Senescence-Associated Secretory Phenotype (SASP)

The process of cellular senescence involves a permanent proliferative arrest with phenotypic changes. Among these changes is the release of several bioactive molecules known as SASPs [34]. In a DTP state, the gene expression characteristics involved in the inflammatory response, epithelial-to-mesenchymal transformation (EMT), and protein secretion are upregulated, whereas the cell cycle-related gene expression characteristics are strongly downregulated [19]. A strong correlation exists between the DTP state and cell senescence. 

The significant enrichment of senescence-related gene expression characteristics in resting cells reveals their similarity to cell senescence [35]. A study has shown that almost half of aging-related genes are expressed in this kind of cell [36]. However, this condition is still not the same as cell senescence, which is an irreversible process of cell death, whereas the DTP state is reversible [20].

In the process of studying the DTP state, the distribution of senescent cells has been found to show differences. The CD133low cell population has indicated SASP and a higher potential for secreting various inflammatory cytokines and chemokines, including interleukin-6, TGF-β, and tumor necrosis factor-α, than the CD133high population [37]. Therefore, CD133 may be the key to imparting a senescence-like phenotype to the DTP state.

Although senescence signatures are observed in the DTP state, to date, their primary role in the formation of the DTP state has not been definitively discovered. Further research is needed to decipher the role of this pseudosenescence phenotype in drug tolerance and drug resistance [22].

### 2.5. Epigenetic Regulation

Epigenetics has always been considered to play an important regulatory role in the occurrence and development of cancer [38]. The production of a DTP state does not involve genetic changes but is related to epigenetics [39]. The study of heritable variations in phenotype and gene expression that are unrelated to the DNA sequence is known as epigenetics. DNA methylation, histone modification, chromatin structure regulation, and non-coding RNA regulation are the primary epigenetic mechanisms [40]. The occurrence of the DTP state is especially closely related to methylation and demethylation.

The KDM family is an important member involved in DTP state demethylation regulation. An initial study found that the establishment of drug tolerance requires histone KDM5A/RBP2/Jarid1A and is the basis for reversible drug tolerance [6]. Histone KDM is responsible for the catalytic removal of methylation marks on histone lysines [41]. KDM5 and KDM2B have been shown to be involved in the epigenetic regulation of the DTP state, and their inhibitors can effectively reduce the survival rate of tumor cells [42,43]. The contribution of KDM5A to the DTP state was found in EGFR-mutant lung and HER2+ breast cancer cells [44]. KDM5D was determined to be upregulated in head-and-neck squamous cell carcinoma, promoting the formation of the DTP state [45]. KDM5B was shown to promote tumor drug resistance in a study of melanoma cells [46].

The methylation regulation of the DTP state cannot be ignored either. One key epigenetic alteration that characterizes heterochromatin in unicellular to multicellular species is histone H3 lysine 9 (H3K9) methylation [47]. Meanwhile, it is commonly known that methylation of histone H3 lysine 27 (H3K27) is a transcriptionally repressive chromatin modification [48]. In the DTP state, H3K9 and H3K27 methylation increase [49]. Histone H3 lysine-4 trimethylation may be the key factor to regulate the transition from the DTP state to a DTEP state [50].

## 3. Similarity between Diapause-like DTP State and Embryonic Diapause

Diapause has proven to be a possible operating mechanism of the DTP state [8]. To date, many similarities have been found between embryonic diapause and the DTP state. Understanding these similarities is helpful to understanding the diapause-like DTP state. The authors will explain the similarities in transcription, biochemical metabolism, and autophagy.

The diapause-like DTP state and embryonic diapause are known to downregulate cellular transcription and translation programs via pivotal pathways, such as mTOR and c-Myc [8,51,52]. Low c-Myc expression levels inhibit hexokinase expression and induce diapause [53]. As diapause enters and exits, differential c-Myc activity orchestrates the complex and rapid changes that occur [52]. A low level of c-Myc is one of the main reasons for the formation of a diapause-like DTP state [54]. mTOR is one of the key molecules that causes embryonic diapause [51]. The epigenetic state of diapause is influenced by mTORC1/2 inhibition, which is regulated by amino acid levels [55]. The low levels of mTOR also cause tolerance-persisting proteins to emerge, resulting in diapause-like conditions [56]. Lkb1, the upstream kinase of AMPK, inhibits mTOR, thereby inducing reversible embryonic diapause [55]. Accordingly, we speculate that it is also beneficial to the formation of a diapause-like DTP state.

With the transition of blastocysts from preimplantation to diapause, glycolysis and cholesterol synthesis are upregulated [55]. Fat decomposition increases because of the inhibition of mTORC2 [57]. This finding is consistent with the findings in a diapause-like DTP state. 

SMC4 attenuation induces high glycolysis activity in a diapause-like DTP state, which leads to an increase in lactic acid production [20]. Abnormal glycolysis, which supports tumor cell survival, plays an important role in tumor drug resistance [58]. Glycolysis induces drug resistance in cancer cells by prompting EMT and autophagy [59]. In fact, glycolysis-related enzymes, substrates, and products, such as lactate dehydrogenase, glucose transporters, and the pyruvate dehydrogenase complex, all participate in these mechanisms [60]. Lactic acid has no nutritional value for humans; however, it is a source of nutrition for tumor cells [61]. High lactate levels increase ABC transporter expression through histone lactylation, making tumor cells minimally sensitive to therapy [20], which may be one of the mechanisms of DTP formation. The value of lactic acid in a diapause-like DTP state needs to be further explored. 

Cancer-associated adipocytes in the TME are the main participants in tumor lipid metabolism, and promoting lipolysis and activating autophagy may be the mechanism of its activation of a diapause-like DTP state [62].

By breaking down intracellular components and supplying cells with degradation products, autophagy, a general term for the physiological process by which cells direct their components to the lysosome via autophagosomes for degradation, is crucial for preserving and regulating cell homeostasis [63]. Autophagy plays an active role in embryonic diapause: that is, it is one of the important mechanisms of embryonic diapause [64]. 

Similarly, autophagy is found in tumor dormancy and drug resistance and may be one of the mechanisms of a diapause-like DTP state. Autophagy is a survival pathway and quality control system that can stop tumorigenesis and slow the spread of cancer in the early stages of tumorigenesis. However, autophagy, a dynamic degradation and circulatory system, contributes to the survival and growth of established tumors and increases cancer invasiveness by boosting metastasis once a tumor has grown, has become established, and is exposed to external pressure [65]. In response to drug therapy, the enhancement of autophagy leads to the emergence of tumor resistance [66]. 

An essential step in the formation of the embryo is the biological process known as EMT, which permits polarized epithelial cells, which typically interface with the basement membrane through their basal surface, to go through a number of biochemical changes and adopt the phenotype of mesenchymal cells [67]. Autophagy can promote or inhibit EMT in accordance with specific conditions, and EMT contributes to the dormancy and drug resistance of tumor cells [68]. The inhibition of mTOR will also cause autophagy, which, in turn, promotes the occurrence of a DTP state [69]. As a serine/threonine kinase, UNC-51-like kinase 1 (ULK1) initiates autophagy in mammals [70]. It is also found in tumors and may be related to tumor dormancy and drug resistance [71,72]. The authors think that ULK1 may also be involved in the formation of a diapause-like DTP state.

## 4. Treatment Strategy of Diapause-like DTP State

Although various therapeutic strategies have been produced on the basis of the life processes in a diapause-like DTP state, they have a common purpose: arbitrarily controlling the life activities in a diapause-like DTP state at any stage of the DTP state to improve the condition of cancer patients. Here is a list of drugs that are resistant to different types of tumor cells and the corresponding improvements that may facilitate the elimination of treatment-persistent residual tumor cells (Table 1). Among them, NSCLC and melanoma are the more resistant cancer types and have been studied more intensively.

### 4.1. Prevention of Diapause-like DTP State Formation

Investigating the mechanism of diapause-like DTP state development and identifying targets to prevent it are crucial therapeutic strategies.

Cotreatment with the PI3K/mTOR inhibitor dactolisib (BEZ235) and the bromodomain and extraterminal domain inhibitor histidyl-tRNA synthetase (JQ1) prevents changes in the open chromatin architecture and inhibits the acquisition of a diapause-like DTP state [80]. The PI3K-mTOR pathway blockade prohibits myeloid cell leukemia-1 (MCL1) upregulation. mTORC2 can bind and phosphorylate MCL1, facilitating its interaction with the Bcl-2-interacting mediator of cell death. Therefore, polytherapies combining PI3K, mTOR, or MCL1 inhibitors with antineoplastic TKIs may be adequate in stopping the formation of a diapause-like DTP state and furthering the eradication of cancer [81]. CD74 upregulation occurs in the diapause-like DTP state in some clusters of H1975 and PC9-EROR cells, and CD74 may contribute to diapause-like DTP state emergence; thus, CD74 inhibition may be a novel strategy to suppress diapause-like DTP state emergence [82]. 

Regulators of H3K9me3-mediated heterochromatin formation contribute to survival in a diapause-like DTP state, and the rise in H3K9me3 in this state is most pronounced over LINE-1. Therefore, LINE-1 may be a target for preventing the formation of a diapause-like DTP state [49].

The osimertinib diapause-like DTP state is often accompanied by the upregulation of NOTCH1 and Notch target genes, so the combination of γ-secretase inhibitor (GSI) (a Notch inhibitor) with osimertinib may avoid the occurrence of a diapause-like DTP state [83].

**Figure 3:**
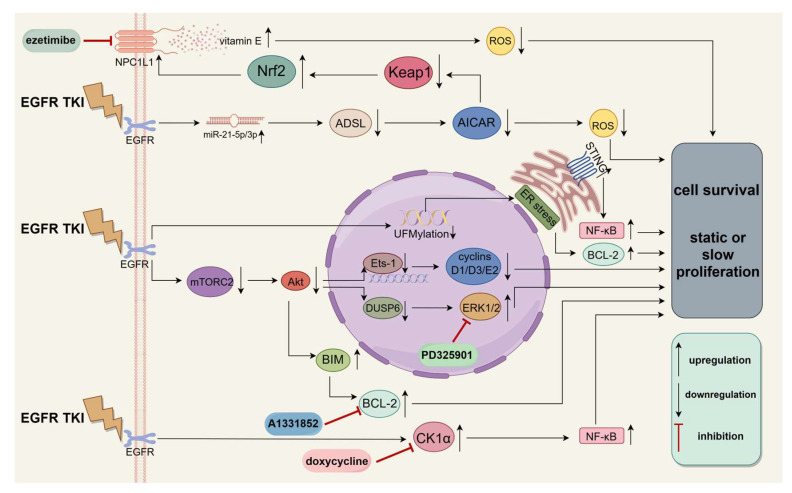
Partial formation mechanism of diapause-like DTP state and some blocking drugs. The diapause-like DTP state tolerant to EGFR TKI is considered as an example. After EGFR TKI treatment, cancer cells enter the diapause-like DTP state through a series of complex mechanisms. The use of CK1α inhibitor doxycycline, NPC1L1 inhibitor ezetimibe, BCL-2 inhibitor A1331852, and ERK1/2 inhibitor PD325901 may prevent cancer cells from entering a diapause-like DTP state. EGFR, epidermal growth factor receptor; TKI, tyrosine kinase inhibitors; NPC1L1, Niemann-Pick C1-like 1; Nrf2, Nuclear factor erythroid2-related factor 2; Keap1, Kelch-like ECH-associated protein 1; ADSL, adenylosuccinate lyase; AICAR, 5-Aminoimidazole-4-carboxamide1-β-D-ribofuranoside; STING, stimulator of interferon gene; NF-κB, nuclear factor kappa-B; BCL-2, B-cell lymphoma-2; Ets-1, E26 transformation-specific-1; mTORC2, mammalian target of rapamycin complex 2; DUSP6, dual-specificity phosphatase 6; and BIM, Bcl-2-interacting mediator of cell death. “↑” indicates upregulation, and “↓” indicates downregulation.

Despite the wide range of potential treatments already accessible, because of DTP’s intricate mechanisms, combination therapies may be more efficient. We summarize some of the mechanisms of formation of a diapause-like DTP state and believe that there will be more treatments to block these mechanisms in the future (Figure 3).

### 4.2. Maintenance of Diapause-like DTP State

Formulating a reasonable treatment in accordance with the mechanism of a diapause-like DTP state is also a promising way of maintaining the diapause-like state and avoid the damage of cancer cells to the human body.

One possible way to treat diapause is to reduce the activity of c-Myc or block cyclin-dependent kinase 9 to maintain the dormancy of the remaining cells after therapy [54].

Similarly, inhibition of mTOR activity is a potential therapeutic strategy. The performance of current mTOR inhibitors, such as rapalogs and TORKi, is unsatisfactory [84]. As a new generation of mTOR inhibitors, RapaLink-1 is a good choice for this treatment strategy [85]. SBI-0206965 is a highly selective ULK1 kinase inhibitor that can be combined with mTOR inhibition to degrade ULK1 and block autophagy, which may be useful in maintaining this therapeutic strategy [86].

Recently, a novel treatment that uses miRNA regulation to keep the diapause-like DTP state dormant was proposed [87]. The treatment seems to be accurate and effective and is worthy of further study. P-glycoprotein (P-gp) is upregulated in a diapause-like DTP state, and, when it is inhibited by the P-gp inhibitor tariquidar, the diapause-like DTP state is prolonged [76]. This treatment strategy needs to be maintained by means of long-term administration and regular testing. However, long-term administration may lead to chronic toxic reactions, which are harmful to patients’ health. Therefore, developing drugs that do little harm to patients’ health is necessary to carry out this treatment.

### 4.3. Withdrawal of Diapause-like State

Inhibition of mTOR and Myc is an important cause of embryo diapause [88]. It also leads to the formation of a diapause-like DTP state [54,69]. Through upregulating their expression, tumor cells may be able to withdraw from the diapause-like DTP state and regain sensitivity to drugs. However, current research on them is focused on inhibitors [89,90]. Their activators require further exploration.

The Wnt Family Member (WNT)-Leucine Rich Repeat Containing G Protein-Coupled Receptor 5 (+) stem cell program is downregulated in the exit stage of a diapause-like DTP state [91], which may also be a target to force cancer cells to withdraw from their diapause-like DTP state. By increasing SFRP1, WNT5A can be inhibited to bring cancer cells out of dormancy [17].

## 5. Perspectives and Conclusions

PGCCs may be a bridge between embryonic diapause and the diapause-like DTP state [92]. Diapause is a dynamic process consisting of several successive phases encompassing induction, preparation, initiation, maintenance, and termination [93]. The life cycle of PGCCs is like that of diapause. The beginning, self-renewal, termination, and stability of the life cycle of PGCCs correspond to the beginning, maintenance, termination, and awakening of embryo diapause, respectively [94]. According to recent findings, PGCCs may have developed an early embryonic-like program that was activated in response to oncogenic and therapeutic stress, reprogramming cancer cells to be resistant to drugs and capable of spreading [95].

Polyploidy caused by chemotherapy was first discovered in 1960 [96]. Since then, increasing evidence indicates that PGCCs are formed under environmental and therapeutic stressors, and they are increasingly emphasized as promising therapeutic targets for overcoming drug resistance and recurrence [97]. PGCCs have the ability to start the metastatic cascade, withstand therapeutic doses that are considered “lethal”, and divide asymmetrically to produce normal cancer cells that are more resistant to various anticancer drugs [98]. The characteristics of PGCCs make them associated with a diapause-like DTP state. These findings indicate that PGCCs are an important model for studying the diapause-like DTP state.

Moreover, single-cell sequencing is an excellent technique for monitoring and tracking cancer cell state and finding heterogeneity [99], which is helpful for us to explore the development of a diapause-like DTP state. For example, fateMap, a framework which combines DNA barcodes with single-cell RNA sequencing, has been developed to study the state changes in cancer cells after therapy [100]. To deal with the emergence of a diapause-like DTP state caused by EGFR inhibitors, we can trace the origin and clonal evolution of drug-resistant cells with high resolutions by combining an expression barcode system and single-cell RNA sequencing to find an effective treatment [101]. Some single-cell sequencing techniques have identified the DTP state well. Arc-well, a high-throughput single-cell DNA sequencing method compatible with formalin-fixed and paraffin-embedding materials, is a good example [102]. Fate-seq, an approach to isolate single cells in their transient states of drug sensitivity or tolerance before profiling, has also been proposed [103]. The authors believe that, in the future, the application of a single-cell sequencing technique will help researchers to find and solve the heterogeneity of the diapause-like DTP state so as to better treat tumor drug resistance.

Here, we emphasize that the biomarkers of the diapause-like DTP state are rare and need to be explored. For example, TEA Domain Transcription Factor and DNA Fragmentation Factor Subunit Beta are potential biomarkers [104,105], but more experiments are needed. Breakthrough in this aspect may greatly promote the identification and treatment of the diapause-like DTP state.

To conclude, the mechanism of the diapause-like DTP state is not perfect, and unraveling its secrets may help researchers understand the phenomenon of tumor dormancy. The study of the diapause-like DTP state is relatively deep. Studying the diapause-like DTP state from the perspective of embryonic diapause rather than from the perspective of tumor dormancy may help researchers understand the diapause-like DTP state. For example, Forkhead Box O and Transcription Factor EB are the core actors in sensing harmful environments and transmitting signals to stimulate the body to enter embryonic diapause [106,107]. Hence, they may also be involved in the formation of a diapause-like DTP state. Overall, the discovery of the diapause-like DTP state opens a new door to the tumor world. The authors believe that the diapause-like DTP state will become an important goal of tumor therapy soon.

## Figures and Tables

**Table 1:** Treatment of different drug-resistant tumors in a diapause-like DTP state.

Tumor Types	Tolerant Drugs	Key Pathways/ Genes	Changes in Key Pathways/Genes in a Diapause-like DTP State	Drugs to Improve Treatment	References
HNSCC	cisplatin	KDM5D/ AURKB	upregulation	barasertib	[45]
Melanoma	BRAF inhibitor and MEK inhibitor	cIAP2	upregulation	birinapant	[73]
Melanoma	BRAF inhibitor and MEK inhibitor	Peroxisomal and UGCG	upregulation	inhibitor of the PEX3–PEX19 interaction and UGCG inhibitor	[74]
Melanoma	BRAF inhibitor and MEK inhibitor	RTK	upregulation	RMC-4550	[75]
NSCLC	EGFR TKI	BIM	upregulation	BH3 mimetics	[39]
TNBC	doxorubicin	P-gp	upregulation	tariquidar	[76]
NSCLC	EGFR TKI	IGF1R	upregulation	cIGF1R	[77]
PDAC	irinotecan	CYP3A	upregulation	ketoconazole	[78]
CLL	venetoclax	Bax/Bak	downregulation	DT-061	[79]

HNSCC, head-and-neck squamous cell carcinoma; BRAF, serine/threonine kinase; MEK, mitogen-activated extracellular signal-regulated kinase; UGCG, UDP-glucose ceramide glycosyltransferase; PEX, Peroxisomal; NSCLC, non-small cell lung cancer; EGFR, epidermal growth factor receptor; TKI, tyrosine kinase inhibitors; TNBC, triple-negative breast cancer; P-gp, P-glycoprotein; IGF1R, insulin-like growth factor 1 receptor; cIGF1R, circular RNA IGF1R; PDAC, Pancreatic ductal adenocarcinoma; CYP3A, cytochrome P450 3A4; CLL, chronic lymphocytic leukemia; Bax, BCL2 associated X apoptosis regulator; and Bak, BCL2 antagonist/killer 1.

## Data Availability

Not applicable.
